# Serine 216 Phosphorylation of Estrogen Receptor α in Neutrophils: Migration and Infiltration into the Mouse Uterus

**DOI:** 10.1371/journal.pone.0084462

**Published:** 2013-12-26

**Authors:** Sawako Shindo, Rick Moore, Gordon Flake, Masahiko Negishi

**Affiliations:** 1 Pharmacogenetics Section, Laboratory of Reproductive and Developmental Toxicology, National Institute of Environmental Health Sciences, National Institutes of Health, Research Triangle Park, North Carolina, United States of America; 2 Laboratory of Pathology, National Institute of Environmental Health Sciences, National Institutes of Health, Research Triangle Park, North Carolina, United States of America; Institut de Génomique Fonctionnelle de Lyon, France

## Abstract

**Background:**

Whereas estrogen receptors are present in immune cells, it is not known if they are phosphorylated to regulate immune cell functions. Here we determined the phosphorylation status of estrogen receptor α (ERα) at residue serine 216 in mouse neutrophils and examined its　role in migration and infiltration. Serine 216 is the conserved phosphorylation site within the DNA binding domains found in the majority of nuclear receptors.

**Methodology/Principal Findings:**

A phospho-peptide antibody specific to phosphorylated serine 216 and ERα KO mice were utilized in immunohistochemistry, double immuno-staining or Western blot to detect phosphorylation of ERα in peripheral blood as well as infiltrating neutrophils in the mouse uterus. Transwell assays were performed to examine migration of neutrophils. An anti-Ly6G antibody identified neutrophils. About 20% of neutrophils expressed phosphorylated ERα at serine 216 in peripheral white blood cells (WBC) from C3H/HeNCrIBR females. Phosphorylation was additively segregated between C3H/HeNCrIBR and C57BL/6 females. Only neutrophils that expressed phosphorylated ERα migrated in Transwell assays as well as infiltrated the mouse uterus during normal estrous cycles.

**Conclusions/Significance:**

ERα was phosphorylated at serine 216 in about 20% of mouse peripheral blood neutrophils. Only those that express phosphorylated ERα migrate and infiltrate the mouse uterus. This phosphorylation was the first to be characterized in endogenous ERα found in normal tissues and cells. Phosphorylated ERα may have opened a novel research direction for biological roles of phosphorylation in ERα actions and can be developed as a drug target for treatment of immune-related diseases.

## Introduction

Inflammation is a critical factor associated with the development of estrogen-dependent diseases including breast cancer [1-3]. The knockout of ERα in NZM2410 and MRL/lpr lupus prone mice reduces symptoms of systemic lupus erythematous and prolongs survival [4]. In addition to response to inflammation, neutrophils also infiltrate tissues under normal physiological conditions; for instance, neutrophils are known to infiltrate the mouse uterus in response to estrogen, migrate and detach into the lumen in response to hormonal cycles [5-7]. When this uterine infiltration occurred in progesterone receptor-null females, estrogen treatment accumulated neutrophils underneath the uterine luminal epithelium and caused inflammatory reactions [5]. ERα is known to act as an essential regulatory factor responsible for these estrogen actions [1]. On the other hand, while estrogen receptors (ERα and ERβ) are known to exist in neutrophils [8], whether or not they play any independent role in neutrophil infiltration during estrous cycle has not been established. Moreover, estrogen receptors may be differently modified from those in uterine cells, thereby directing their response to infiltration. Here we have focused on ERα and examined phosphorylation of ERα in mouse neutrophils and its role in migration and infiltration.

Although ERα is reported to be phosphorylated in tumor tissues and transformed cells such as MCF7, phosphorylation of endogenous ERα has not been convincingly demonstrated in normal tissues [9,10]. Nuclear constitutive active/androstane receptor (CAR, NR1I3) belongs to the nuclear steroid hormone superfamily which includes ERα. CAR is activated by various therapeutic drugs such as the anti-epileptic drug phenobarbital. Unlike estrogen that directly binds to ERα to activate it, phenobarbital indirectly activates CAR through de-phosphorylation of CAR at threonine 38 [11,12]. Threonine 38 is located in the region between the two zinc fingers within the DNA binding domain (DBD) of the CAR molecule and constitutes a phosphorylation site by protein kinase C. Amino acid sequence alignments reveal that this phosphorylation motif is conserved in the majority of nuclear receptors. ERα conserves this motif and residue as serine 212 and serine 216 in the DBD of human and mouse receptors, respectively. Mutation studies of serine 212 to alanine and aspartic acid found phosphorylation mimicking ERα S212D mutant regulates a distinct set of the genes from the non-phosphorylation mimicking ERα S212A mutant in hepatoma-derived Huh7 cells [13]. Given these findings, here we have utilized an antibody (designated αP-S216) that specifically detects phosphorylation of serine 216 of ERα and examined whether endogenous ERα is phosphorylated in normal mouse tissues and cells.

In our present studies, we first employed αP-S216 to immunohistochemically screen various tissues from C3H/HeNCrIBR female mice for phosphorylation. The resulting strong staining of some cells in the uterus prompted us to further investigate these cells. Western blot analysis revealed the presence of phosphorylated and non-phosphorylated ERα in the mouse uterus. Double fluorescence staining with a neutrophil-specific Ly6G antibody confirmed that αP-S216 antibody stains infiltrating neutrophils. Similar staining revealed that a fraction of mouse peripheral blood neutrophils expressed phosphorylated ERα. Moreover, mouse white blood cells were prepared from C3H/HeNCrIBR as well as ERα KO females to examine the role of phosphorylated ERα in the migration of neutrophils using a Transwell system. Here we present experimental results to discuss the hypothesis that phosphorylated ERα may elicit a distinct signal to confer blood neutrophils with spontaneous migration as well as infiltration capabilities into the mouse uterus. 

## Results

### Phosphorylation of ERα at serine 216 in infiltrating neutrophils in the uterus

**Figure 1:**
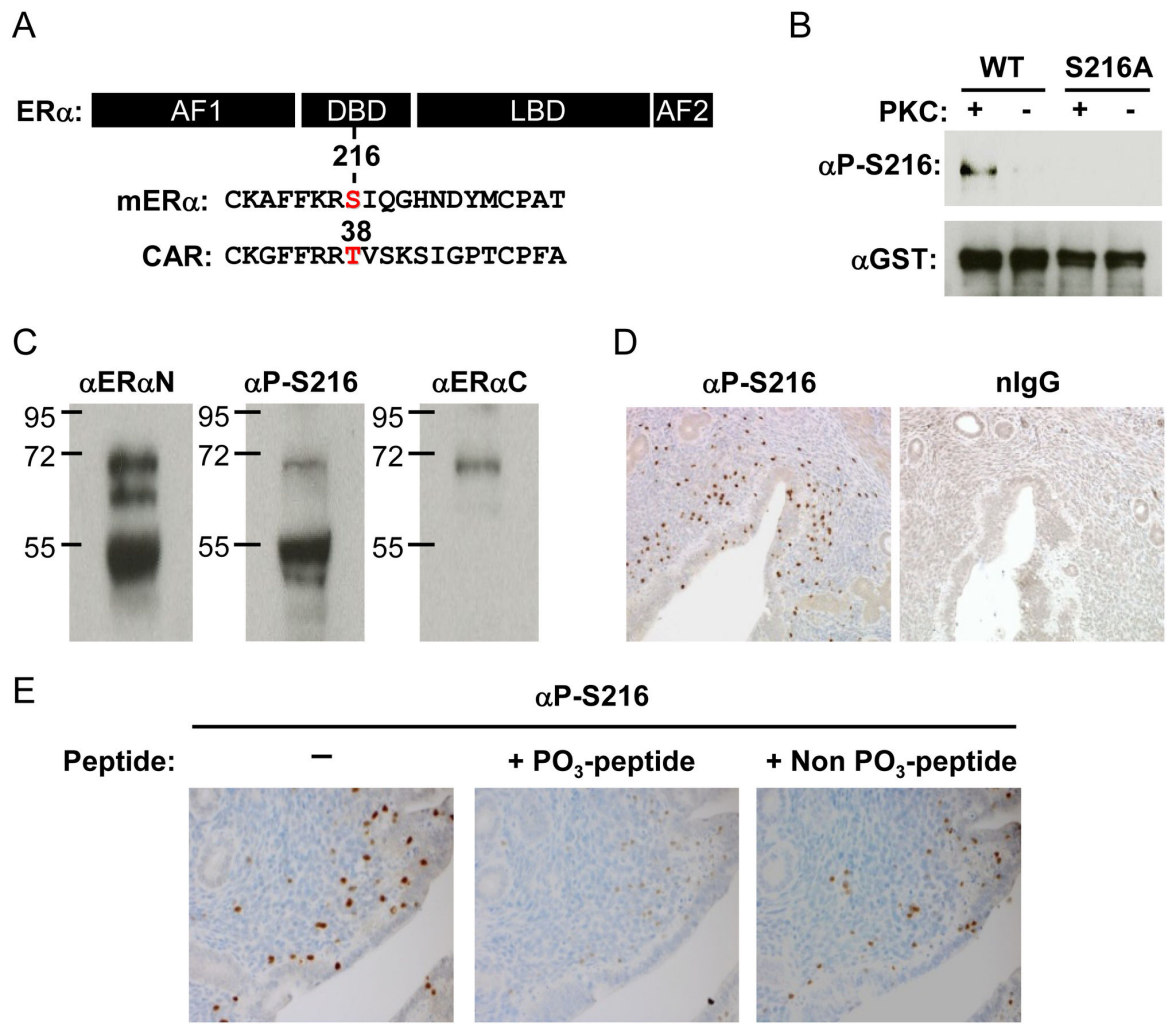
Phosphorylation of ERα at serine 216. (A) Map to show localization of serine 216 in ERα and threonine 38 in CAR. (B) *In* *vitro* phosphorylation of serine 216. Purified glutathione S transferase (GST)-mERα wild type (WT) and GST-S216A were incubated with or without protein kinase C (PKC) and Western blots were performed using an anti-P-Ser-216 (αP-S216) or anti-GST antibody. (C) Western blot analysis of whole extracts (20 μg protein/well) prepared from the mouse uterus at the estrus stage with αP-S216 or an anti-ERα antibody (αERαN or αERαC). (D) Uterine sections were immune-stained using αP-S216 antibody or normal IgG. (E) Uterine sections to determine staining specificity of αP-S216. αP-S216 antibody was mixed with phospho- or non-phospho-peptides prior to incubation with sections.

Serine 216 in mouse ERα is conserved as a phosphorylation motif similar to that of threonine 38 in the nuclear receptor CAR (Figure 1A). First, Western blot analysis with an αP-S216 antibody confirmed that serine 216 could be phosphorylated by protein kinase C utilizing purified GST fused recombinant GST-ERα protein (wild type or its dominant negative mutant) in *in vitro* kinase assays (Figure 1B). Equal presence of recombinant ERα proteins in the reactions was verified by Western blot with a αGST antibody. Whole extracts were prepared from the mouse uterus at the estrus stage and subjected to Western blot analysis (Figure 1C). The αERαN antibody (against the N- terminal region of ERα) detected full length ERα and two truncated forms. The two truncated forms, with approximate molecular weights 62 k and 55 k Daltons), appear to be products of degradation generated during sample preparation and lacked the C-terminal region since an αERαC antibody (against the C-terminal region) did not bind to these truncated forms and bound to only full length ERα. The αP-S216 antibody reacted with full-length ERα and the third truncated form that should include serine 216 based on its 55 kDa molecular weight. On the other hand, αP-S216 did not bind the second truncated form which was primarily generated from non-phosphorylated ERα, thereby suggesting the presence of both phosphorylated and non-phosphorylated ERα in the mouse uterus. Subsequent immunohistochemistry with αP-S216 antibody revealed that a number of, but not all, stained cells are scattered in the uterus (Figure 1D). Specificity of this staining was confirmed by immunohistochemistry in the presence of competing phosphorylated or non-phosphorylated peptides (Figure 1E). Thus, phosphorylated ERα appeared to be expressed in only a distinct type of cell in the uterus.

**Figure 2:**
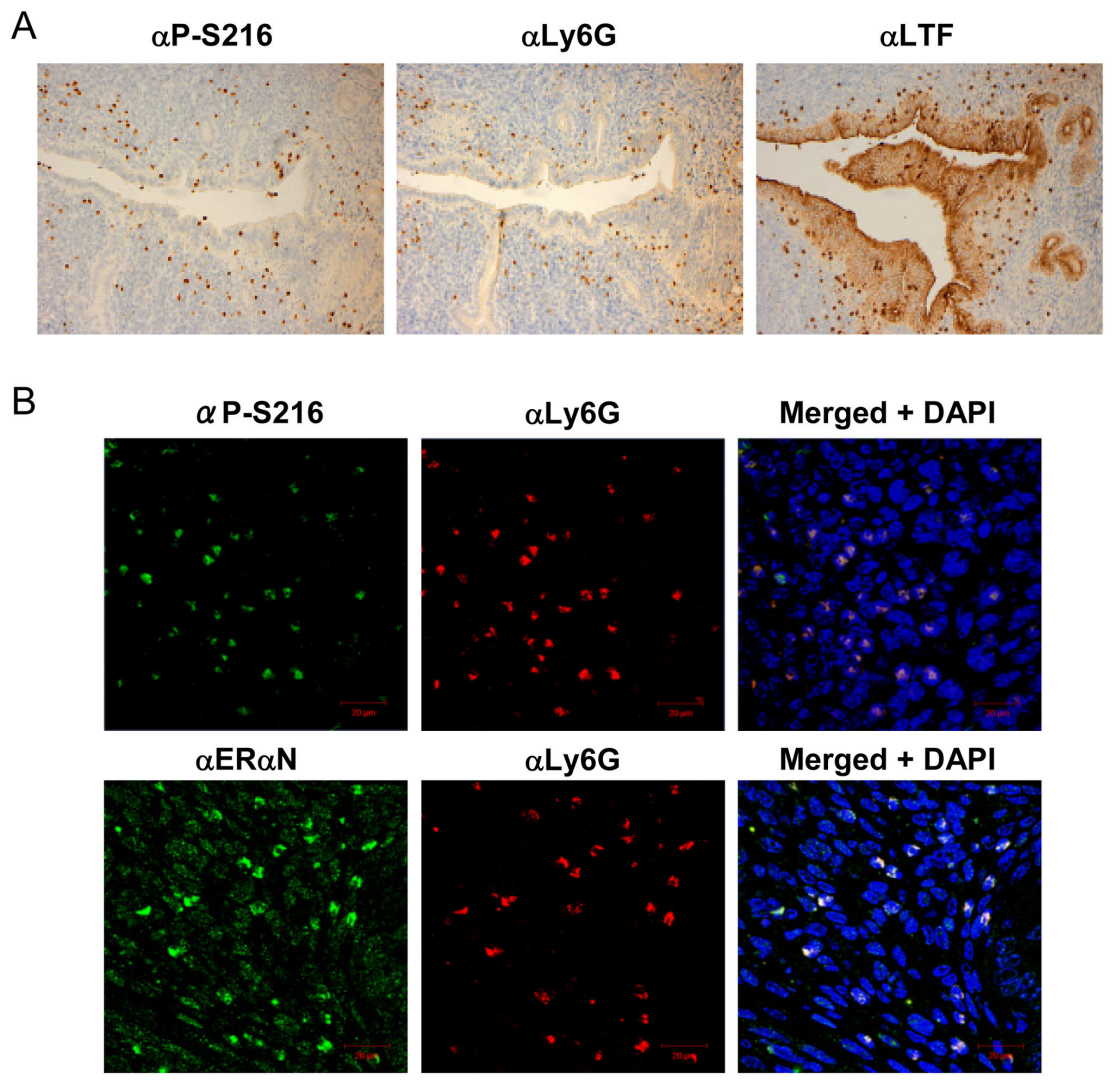
Phosphorylated ERα in infiltrating neutrophils. (A) Serial sections of the uterus at the estrus stage were stained by αP-S216, anti-Ly6G (αLy6G) or anti-lactoferrin (αLTF) antibody. (B) Uterine sections were double stained with αLy6G antibody and either αP-S216 or αERαN antibody. αLy6G was visualized by Texas red-conjugated second antibody; while fluorescein-conjugated antibody was used to visualize αP-S216 and αERαN. DAPI stains nuclei.

**Figure 3:**
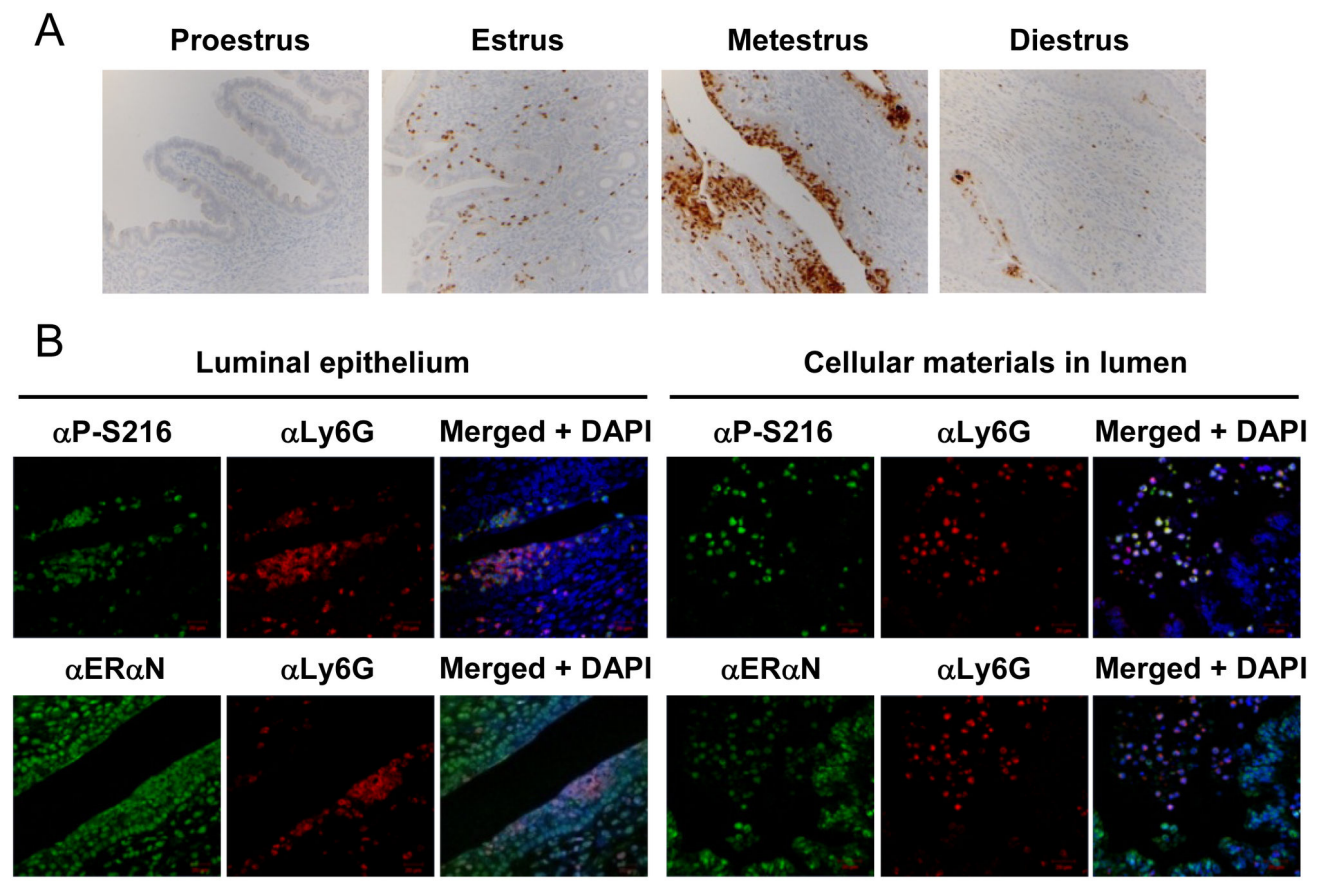
Phosphorylated ERα-expressing neutrophils during the estrous cycles. (A) Mouse uterine sections (at the proestrus, estrus, metestrus or diestrus stage) were stained by an anti-Ly6G antibody (αLy6G). (B) Sections were prepared from the mouse uterus at the metestrus stage and subjected to fluorescence double staining with αLy6G and either αP-S216 or αERαN. Immunoreactions were visualized as described in the legend of Figure 2. DAPI stains nuclei.

Because of this distinct staining pattern by αP-S216, serial sections of the mouse uterus were stained with αP-S216, αLy6G (a specific membrane marker for neutrophils) [14] or lactoferrin (αLTF) antibodies. Lactoferrin is also expressed in neutrophils as well as in uterine luminal and glandular epithelial cells [15,16]. αP-S216 antibody appeared to stain the same cells which were also stained by αLy6G or αLTF antibody (Figure 2A). The αLTF antibody also stained the epithelial cells in addition to those stained by αP-S216 and αLy6G antibodies. Subsequent double fluorescence staining of the stroma region at the estrus stage confirmed that both αP-S216 and αERαN antibodies stained the cells stained by αLy6G antibody (Figure 2B). Thus, the cells stained byαP-S216 antibody were neutrophils that infiltrated the mouse uterus. As expected, in addition to neutrophils, ERαN antibody also stained the nuclei (in oval shapes) of the uterine cells. Immuno-staining with αLy6G antibody of the mouse uterus at the different stages of the estrous cycles demonstrated that neutrophils first infiltrate the stroma during the estrus stage and migrate towards the luminal epithelium and then detach into the lumen as the normal estrous cycle progresses (Figure 3A). Those neutrophils accumulated in the luminal epithelium or detached in the lumen double stained by αP-S216 and αLy6G antibodies (Figure 3B). In addition, double staining was also performed on mouse uterine sections at the proestrus, estrus, metestrus and diestrus stages. Phosphorylated ERα remained expressed at all four stages and no stage-dependent expression of phosphorylated was observed (data not shown). 

### Phosphorylated ERα in peripheral blood neutrophils

**Figure 4:**
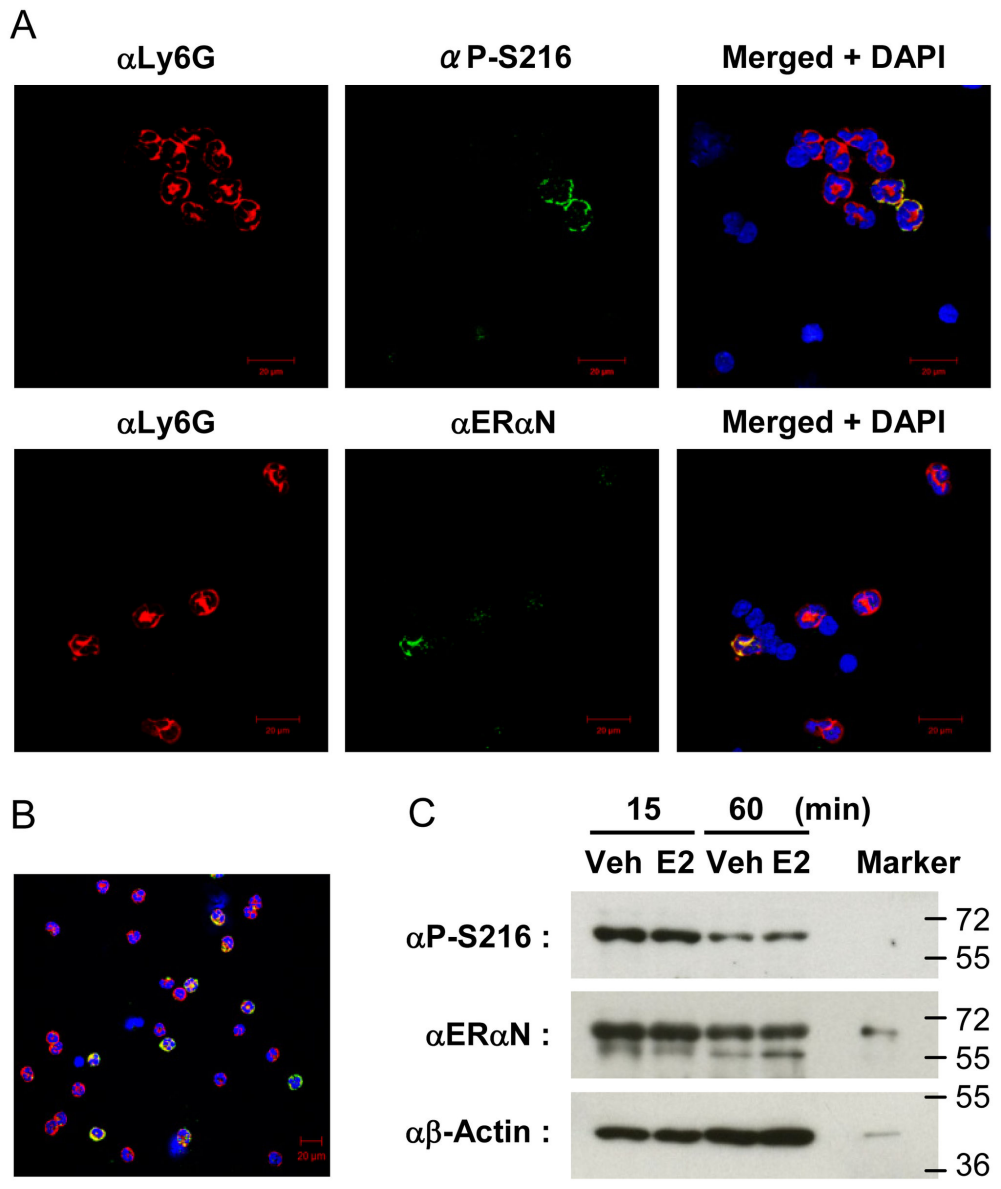
Phosphorylated ERα in peripheral blood and peritoneal neutrophils. (A) WBC were isolated from peripheral blood collected from female mice for double staining with an anti-Ly6G antibody (αLy6G) and either an anti-P-Ser-216 (αP-S216) or an anti-ERαN (αERαN) antibody. Staining and nuclei were visualized as described in the legend of Figure 2. (B) Peritoneal neutrophils were prepared on slides as described in the Materials and Methods section and double stained by αLy6G (in red) and that by αP-S216 (in green). This picture overlaps the double antibody staining with DAPI staining. (C) Peritoneal Neutrophils were cultured with ethanol (vehicle) or estradiol (E2, 10 nM) for 15 or 60 min at 37 °C, from which whole extracts were prepared for Western blots with αP-S216, αERαN or anti-β-Actin (αβActin) antibody. Whole extracts that contained recombinant human ERα (not phosphorylated) was utilized as an ERα marker. αβActin staining endogenous actin was utilized to verify equal amounts of proteins in each well.

Since neutrophils infiltrate from the blood stream into the mouse uterus, WBC fractions were isolated from the mouse peripheral blood of C3H/HeNCrIBR females and subjected to double fluorescence staining using αLy6G and αP-S216 antibodies. Among the eight neutrophils stained by αLy6G antibody on this slide, only two were stained by αP-S216; αERαN antibody stained only one of five neutrophils on the other slide (Figure 4A). Further counting of over 100 to 1,000 neutrophils on a glass slide for each double staining revealed that ERα is expressed in only approximately 20% of blood neutrophils and these ERα are all phosphorylated. Double staining that we performed with WBC fractions that were prepared from at each of the four stages confirmed that the expression levels of phosphorylated ERα remained constant in peripheral blood neutrophils during estrous cycles (data not shown).

Subsequently, peritoneal neutrophils which infiltrated the mouse abdomen after casein injection were utilized to further investigate phosphorylation of ERα. First, double staining found that an approximately 20% of peritoneal neutrophils express phosphorylated ERα as observed with neutrophils in WBC (Figure 4B). To examine whether or not estrogen regulated phosphorylation of ERα in peritoneal neutrophils, they were cultured with or without estradiol, from which cell extracts were prepared for subsequent Western blot analysis. The expression ratios of phosphorylated to total ERα remained constant after estradiol treatment (Figure 4C). These results indicated that phosphorylation was constitutive in infiltrated peritoneal neutrophils and no longer regulated by estradiol under the experimental conditions used. It remains a question as to what mechanism is utilized to decrease ERα levels during the incubation time.

**Figure 5:**
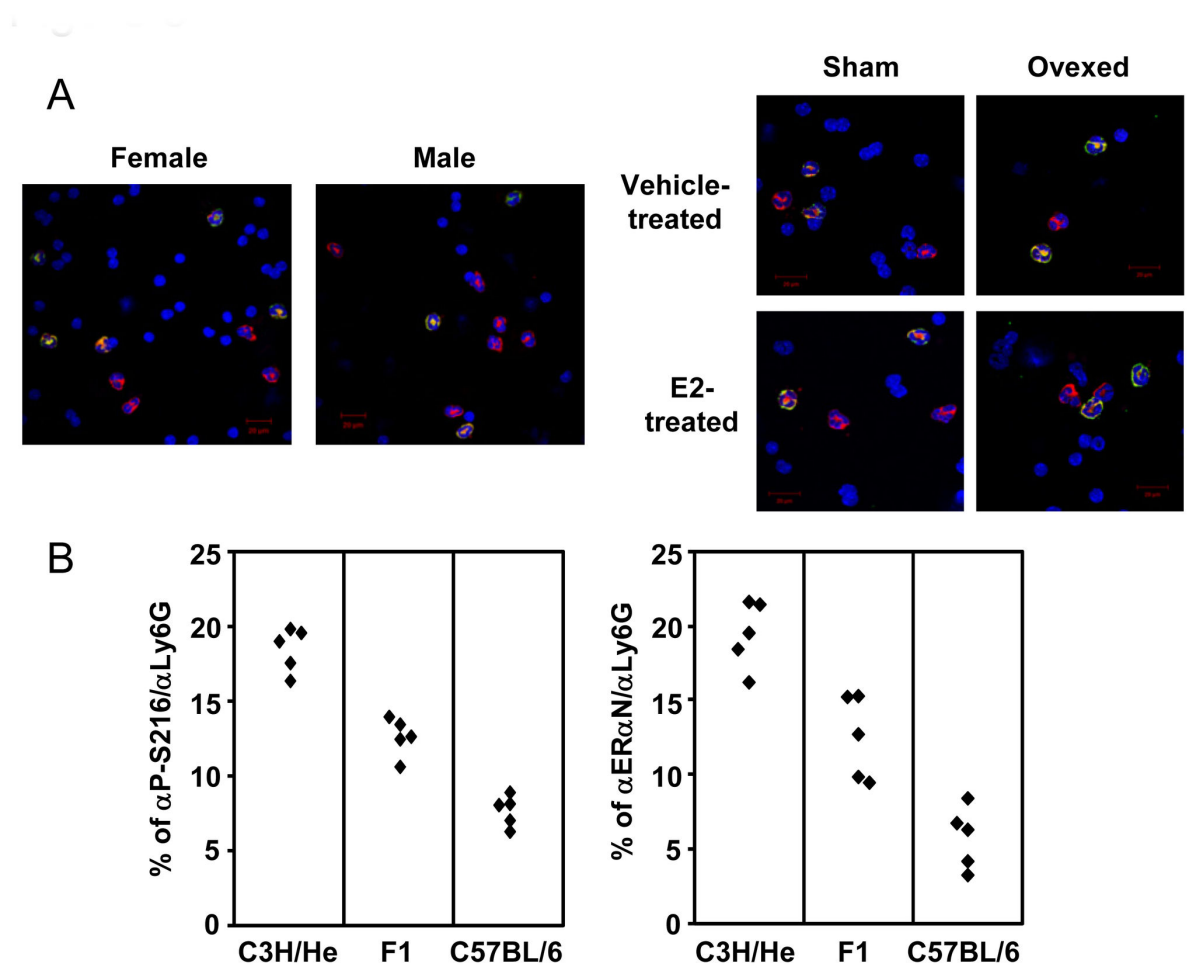
Regulation of ERα phosphorylation in neutrophils. (A) WBC fractions were isolated from C3H/HeNCrIBR females and males and sham-operated and ovariectomized C3H/HeNCrIBR females after treatment with vehicle or estradiol (E2). Pictures present triple-stained cells with αLy6G (in red), αP-S216 (in green) antibodies and DAPI (in blue). (B) Levels of phosphorylated ERα-expressing blood neutrophils in C3H/HeNCrIBR, C57BL/6 and their F_1_ progeny. WBC fractions were isolated from peripheral blood collected from each of five females for a given group and double stained with αLy6G and either αP-S216 or αERαN. Cells double-stained with αP-S216 or αERαN were counted and graphed as percentages of the total neutrophils stained by αLy6G. These numbers counted for staining with αP-S216 were 2000, 200 and 700 for C3H/HeNCrIBR, C57BL/6 and their F1 progeny, respectively, and 2000, 200 and 700 for staining with αERαN. The percentage of αP-S216 antibody-positive cells to total neutrophils was 18.5 ± 0.6. The corresponding percentages were 7.7 ± 0.4 and 12.6 ± 0.5 in blood neutrophils from C57BL/6 and F1 females, respectively. Those percentages of αERαN-positive neutrophils were nearly identical to those obtained for phosphorylated ERα with αP-S216 antibody: 19.5 ± 1.0, 5.9 ± 0.9 and 12.6 ± 1.2 for C3H/HeNCrIBR, C57BL/6 and F1 progenies, respectively. Significances were P <0.0001 and P=0.0002 between C3H/HeNCrIBR and C57BL/6 and C57BL/6 and F1, respectively, for αP-S216 antibody-positive cells. The corresponding values for αERαN antibody-positive cells were P<0.0001 and P=003 between C3H/HeNCrIBR and C57BL/6 and C57BL/6 and F1, respectively.

Phosphorylated ERα-expressing neutrophils were examined in WBC fractions prepared from females, males and overiectomized females (Figure 5A). The levels of ERα-expressing neutrophils remained 20% in all of these WBC fractions. Thus, neither estrogen nor sex altered phosphorylation of ERα in blood neutrophils (Figure 5A). In addition, either hypophysectomy, pregnancy, diabetogenic (ob/ob mice) or inflammation (MRL/lpr lupus prone mice) altered phosphorylation (data not shown). On the other hand, phosphorylation was tightly associated with mouse strain difference between C3H/HeNCrIBR and C57BL/6 females, being additively segregated (Figure 5B). Neutrophils may genetically integrate phosphorylation during development and differentiation. 

### Migration of phosphorylated ERα-expressing neutrophils

**Figure 6:**
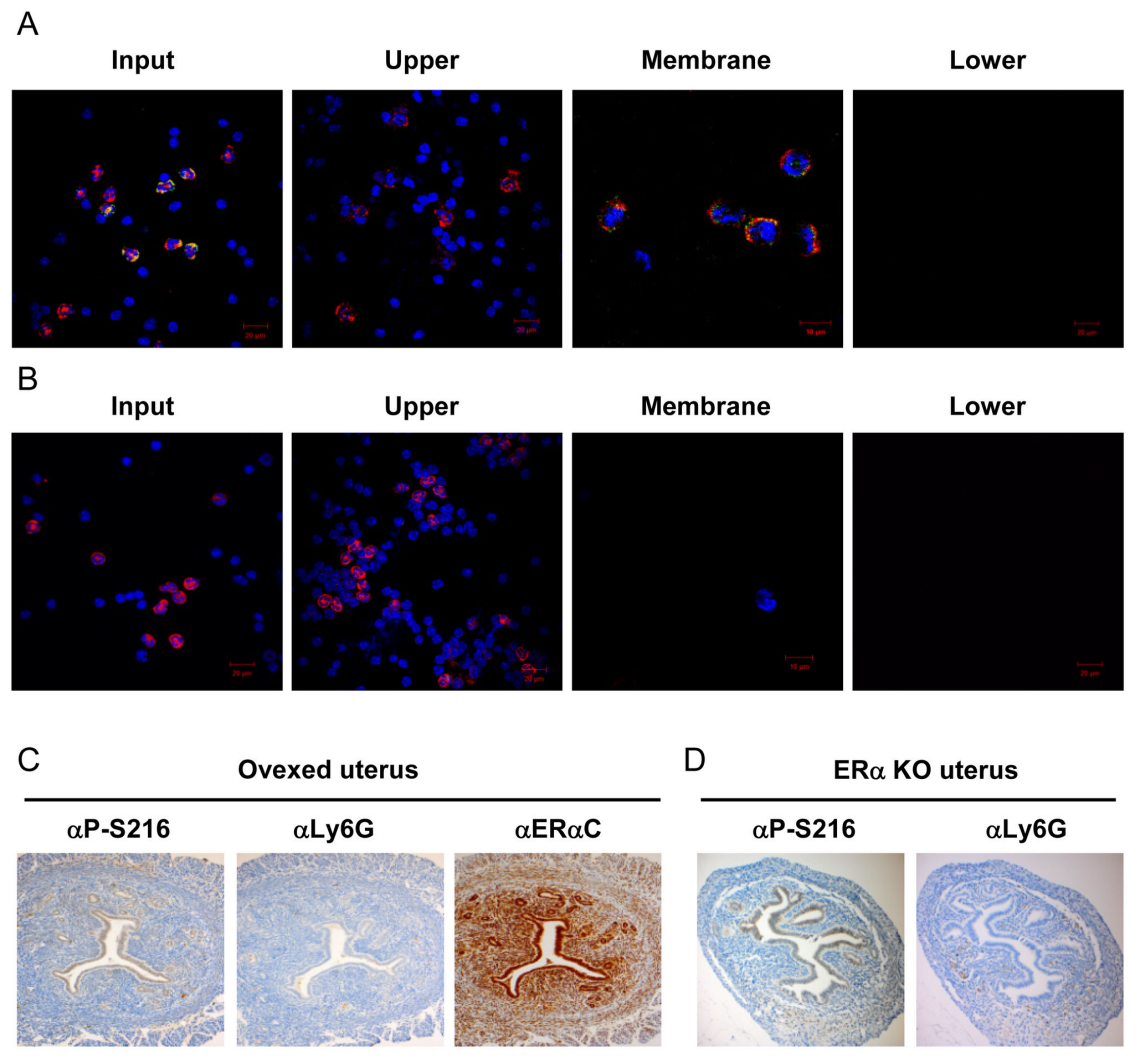
Migration and infiltration of phosphorylated ERα-expressing neutrophils. WBC fractions were prepared from peripheral blood of C3H/HeNCrIBR (A) and ERα KO (B) females and were subjected to migration assays as described in the Experimental section. Cells before migration and cells in upper, lower compartments and the lower surface of the dividing membrane were double stained with anti-Ly6G (αLy6G) and anti-P-Ser-216 (αP-S216) antibodies. Staining was visualized as described in the legend of Figure 2 and images are presented as described in that of Figure 4. (C) Serial sections of the uterus of ovariectomized females were stained by αP-S216, αLy6G or anti-ERαC (αERαC) antibody. (D) Serial sections of the uterus of ERα KO females were stained by αLy6G or αP-S216 antibody.

Given our finding that only 20% of blood neutrophils expressed phosphorylated ERα, while only phosphorylated ERα-expressing neutrophils infiltrated the mouse uterus, we examined whether or not phosphorylation regulated the ability of blood neutrophils to infiltrate. First, we examined the ability of blood neutrophils to migrate in *in vitro* assays. For this end, WBC prepared from C3H/HeNCrIBR females was added to the upper compartment of a Transwell for migration assays (Figure 6A). After the Transwell was left standing for 90 min to allow neutrophils to migrate, neutrophils in upper and lower compartments and on lower surface of dividing membrane were subjected to double staining with αP-S216 and αLy6G antibodies. In over 700 neutrophils counted, 91% of neutrophils were recovered in the upper compartment, which were not stained by with either αP-S216 (Figure 6A) or αERα antibody (data not shown). In contrast, only αP-S216 positive neutrophils were found on the lower surface of the dividing membrane. No neutrophils were detected in the lower compartment. Thus, these results indicated that phosphorylated ERα-expressing neutrophils migrate towards the lower surface of the dividing membrane. This migration was not changed in the presence of estradiol (data not shown). Similar migration assays were performed with WBC prepared from ERα KO female mice. As expected, αERα and αP-S216 antibodies did not stain these neutrophils, nor did those neutrophils migrate, thereby indicating that neutrophils were unable to migrate without the presence of ERα (Figure 6B).

Unlike the case of neutrophils that infiltrated the mouse uterus, both phosphorylated and non-phosphorylated ERα infiltrated the mouse abdomen in response to casein injection (Figure 4B). Both types of blood neutrophils migrated in Transwell assays; before or after migration, the ratios between phosphorylated and non-phosphorylated ERα-expressing neutrophils remained constant (about 20%) (Figure S1). Thus, phosphorylated ERα appeared to be an essential regulator for blood neutrophils to migrate and infiltrate under physiological conditions such as uterine infiltration, but may not be required in inflammatory migration and infiltration, such as those induced by casein injection.

### ERα-dependent infiltration

Although estrogen did not regulate phosphorylation of ERα in blood neutrophils (Figure 5A), the neutrophil infiltration that occurred in the mouse uterus at the estrus stage was strongly indicative of estrogen involvement (Figure 3A). In fact, staining of serial sections of the mouse uterus of ovariectomized C3H/HeNCrIBR females showed that neutrophil infiltration was estrogen dependent (Figure 6C). Furthermore, no infiltration was observed in the uterus of ERα KO females (Figure 6D). Thus, ERα was required for blood neutrophils to infiltrate the mouse uterus in response to estrogen. 

## Discussion

Here we found that serine 216 of endogenous ERα is phosphorylated in mouse neutrophils. Serine 216 is the conserved phosphorylation motif within nuclear receptor super family members. Phosphorylation of serine 216 with ERα in mouse neutrophils was the first to be confirmed with endogenous ERα in normal tissues and cells, and ERα is the second nuclear receptor after CAR in which phosphorylation of the conserved motif has been found. We also demonstrated that only phosphorylated ERα-expressing neutrophils migrate and infiltrate the mouse uterus. Phosphorylated ERα-expressing neutrophils passed the constitutive ability to migrate in the absence of estrogen in an *in vitro* assay. This migration mechanism may engage in diverse regulations depending on type of infiltrated tissue as well as the type of infiltration: inflammatory, hormone dependent or resident.

Phosphorylation of ERα in blood neutrophils was not regulated by estrogen but appeared to be genetically integrated during development of neutrophils. On the other hand, phosphorylated ERα was required in for neutrophils to infiltrate the mouse uterus in response to estrogen since no neutrophils were found in either the ERα KO or overiectomized uterus. The uterus is known to release cytokines to facilitate neutrophil infiltration in response to estrogen [17]. What remains as a critical aspect for the future investigations is the role played by phosphorylated ERα in neutrophils in this uterine infiltration. Neutrophils retained phosphorylated ERα throughout the processes of an intra-uterine migration and of detachment. Moreover, neutrophils are essential for the breakdown and repair of uterine tissues following detachment [18]. The ability of phosphorylated ERα to regulate infiltrating neutrophils in the absence of estrogen may be utilized to participate in the regulation of these processes.

The stimulus that activates phosphorylated ERα and the signal that elicits migrate and/or infiltrate of neutrophils remains unexplored at this time. Phosphorylated ERα was expressed in the cell membrane/cytoplasm of neutrophils before migration (Figures 4 and 5). After migration the neutrophils appeared to sort phosphorylated ERα into the cell membrane, as indicated by a clear co-localization with Ly6G, a membrane surface protein (Figure 4A). Since it is known that estrogen can rapidly elicit non-genomic signaling through membrane ERα [19], this membrane sorting during migration raises the hypothesis that phosphorylated ERα elicits a non-genomic signaling to enable neutrophils to migrate. Despite various inherent experimental limitations with functional analysis of phosphorylated ERα at the molecular level, neutrophils may provide us with an excellent experimental system for future investigations to characterize non-genomic actions of ERα and their mechanisms in the estrogen-dependent infiltration into the uterus.

Neutrophil’s accumulation and detachment during metestrus is thought to resemble what is observed at the peak of decidualization and implantation during pregnancy [20,21]. Infiltrating neutrophils store pro-matrix metalloproteinase 9 in one of its granules and rapidly degranulates pro-enzyme to increase protease activity as numbers of neutrophils increase in the luminal epithelium after mating [6]. Accumulated neutrophils in response to semen exposure were, in fact, activated during implantation. Similar neutrophil activation was also reported to occur in the uterus of progesterone receptor-null mice after estrogen treatment as indicated by the lack of lactoferrin in accumulated neutrophils [5]. However, our study indicates that infiltrating neutrophils do not appear to be activated in the uterus during normal estrous cycles, because lactoferrin was still retained in accumulated neutrophils in luminal epithelial layers as well as those that detached in the lumen at the metestrus stage (Figure S2). It will be of interest to investigate whether phosphorylated ERα plays any role in the activation of neutrophils during pregnancy as well as its mechanisms. For this, future investigations must develop novel animal models such as KI/KO mice to mutate serine 216 to alanine and the neutrophil-specific deletion of this mutated ERα gene and utilize them in combination with known animal models for disease and/or pregnancy.

In summary: Both estrogen and ERα enable blood neutrophils to infiltrate the mouse uterus. This infiltration ability correlated with the presence of phosphorylated ERα at serine 216 in neutrophils. In addition to neutrophils, mouse macrophages were also found to express phosphorylated ERα. Further, peroxisome proliferator-activated receptor was also phosphorylated at its conserved phosphorylation motif (threonine 136) in WBC (unpublished observation). Therefore, this phosphorylation of the conserved motif may be common to nuclear receptors in immune cells and could be the underlying mechanism which regulates these cells in response to endogenous stimuli. Moreover, once ligands specific to phosphorylated nuclear receptors are identified they should provide us with drug candidates that target nuclear receptors in immune cells.

## Materials and Methods

### Materials

An antibody against an anti-phospho-S216 peptide of ERα (αP-S216) and both phosphorylated and non-phosphorylated antigen peptides were produced and evaluated by AnaSpec Inc (San Jose, CA). ERα and lactoferrin antibodies (sc-543, sc-7207 and sc-14434, respectively) were purchased from Santa Cruz Biotechnology (Dallas, TX); a Ly6G antibody (557445) from BD Pharmingen^TM^　(San Jose, CA); VECTASTAIN^®^ ABC KIT (PK-6101 and PK-6104), fluorescein avidin DCS, Texas Red^®^ avidin DCS, avidin/biotin blocking kit and VECTASHIELD^®^ (H-1200) from Vector Laboratories (Burlingame, CA); ExtrAvidin-peroxidase and hematoxylin from Sigma-Aldrich (St Louis, MO); 3, 3’-diaminobenzidine from Dako Cytomation (Carpinteria, CA); estradiol from Steraloids (Newport, RI); Transwell from Corning Incorporated Life Sciences (Lowell, MA).

### Animals and Treatments

C3H/HeNCrIBR, C57BL/6 and their F1 mice (6-7 weeks old) purchased from Charles River Laboratories (Raleigh, NC), were housed and utilized under guidelines approved by the National Institute of Environmental Health Sciences. To obtain female mice at the different stages of the estrous cycle, they were individually housed for 5-7 days and subjected to vaginal smear assays. Estrous cycle stages were determined by H&E staining of vaginal smears. Ovariectomized female mice were fed with standard NIH-31 chow for 3 weeks to clear endogenous ovarian steroids before starting daily treatment with sesame oil or estradiol (0.25 μg/ mouse) for 3 consecutive days. Ex3-ERα KO mice in C57BL/6N back ground [22] were kindly provided by Dr. Kenneth Korach. All protocols and procedures were approved by the National Institutes of Health Animal Care and Use Committee and were in accordance with National Institutes of Health guidelines.

### Immunohistochemistry

Mouse tissues were fixed in 10% formalin, embedded in paraffin and cut to make sections (6 μm thick). Sections were decloaked in a decloaking chamber blocked with 3% H_2_O_2_ for 15 min, incubated in TBS buffer (50 mM Tris-HCl pH 7.4 and 150 mM NaCl) containing normal serum for 1 hr and washed with TBS-T (20% Tween-20 in TBS), treated with a given primary antibody and subsequently incubated with a given biotinylated second antibody for 15 min at room temperature. The sections were treated with ExtrAvidin-peroxidase (50-fold dilution in PBS buffer) for 20 min, incubated with 3, 3’-diaminobenzidine to develop a color signal, counter-stained with hematoxylin, dehydrated and cover-slipped. 

### Preparation of WBC and peritoneal neutrophils

WBC fractions were prepared from blood collected from the inferior vena cava of mice. After adding sodium citrate (for a final concentration of 0.38%), blood was mixed gently and centrifuged at 1,200 rpm for 20 min, from which lower layer was mixed with one fourth volume of pre-warmed 6% dextran and final 0.45% sodium chloride, gently inverted and allowed to stand for 0.5-1 hr. The resulting supernatant was collected as a WBC fraction. Peritoneal neutrophils were induced by intraperitoneal injection with 2 mL of 12% casein (Sigma) for 5 hr. Then, ice-cold PBS containing 1 mM EDTA (10 mL) was injected into the peritoneal cavity and peritoneal fluid was harvested, from which neutrophils were recovered by centrifugation at 960 rpm for 10 min and washed twice using PBS and further purified using CD11b microbeads and AutoMACS (Miltenyi Biotec Inc.).

### Double fluorescence staining

Tissue sections were prepared on glass slides as described under Immunohistochemistry. Mouse WBC fractions were centrifuged onto glass slides using a Cytospin (Thermo Scientific), fixed in 4% formalin, blocked with 0.3% H_2_O_2_, avidin, biotin and finally a normal serum in PBS buffer each for 20 min. For first staining, slides were incubated with a given primary antibody and subsequently with a secondary biotinylated anti-rabbit antibody for 30 min. The secondary antibody was reacted with fluorescein avidin DCS for 8 min. For second staining, these slides were washed with PBS buffer, blocked and incubated with a Ly6G antibody, followed by incubations with a biotinylated anti-rat antibody and then with Texas Red^®^ avidin DCS. Between each of these stainings, sections were washed with PBS buffer and mounted with mounting medium (VECTASHIELD^®^) containing DAPI. 

### Western blots

Frozen mouse uteri or neutrophils were homogenized with a Polytron in TBS buffer containing 8 M urea and 1% SDS and sonicated. Western blot was performed as previously described [13]. 

αERαN and αERαC antibodies were diluted in TBS-T containing 5% skim milk, while αP-S216 antibody was diluted in TBS-T containing 1% BSA.

### Migration assays

WBC obtained from C3H/HeNCrIBR and Ex3 ERαKO females were suspended in RPMI1640 medium containing 3% dextran charcoal stripped serum (Gemini Bio-Products), 2 mM L-glutamine, penicillin (100 U/mL) and streptomycin (100 μg/mL). ). 2 x 10^6^ WBC were added to the upper compartment of a Transwell (3 μm pore size), while the lower compartment was filled with RPMI1640 medium or RPMI1640 medium without serum. Estradiol (10 nM) or vehicle (ethanol) was added to the medium of the lower chamber. After standing for 90 min at 37 °C, cells in both compartments were collected and were fixed onto slides for double fluorescence staining. After the upper surface of the dividing membrane was washed with PBS, the membrane was double stained to observe cells on the lower surface by confocal microscopy.

## Supporting Information

Figure S1 **Inflammatory migration assays (**A**) WBC fractions obtained from C3H females were subjected to migration assay using a Transwell.** The lower compartment was filed with the supernatant of casein-injected peritoneal fluids. After 90 min, cells collected in the lower compartment were fixed onto slides, stained by H&E or double stained by an anti-Ly6G and anti-ERαN antibodies which were visualized by either Texas red-conjugated or fluorescein-conjugated secondary antibody, respectively. Pictures were two regions of a double-stained slide. Both ERα- and non-expressing neutrophils migrated in response to the fluid. The same migration was also obsered in the absence of peritoneal fluid and also to the lower surface of the dividing membrane.
(TIF)

Figure S2 **Expression of lactoferrin in neutrophils in the uterine luminal epithelium and lumen (**A**) Fluorescence double staining of the uterine luminal epithelium at the metestrus stage with anti-lactoferrin (αLTF) and anti-Ly6G (αLy6G) antibodies.** Both Ly6G and LTF are markers for neutrophils. αLTF was visualized by fluorescein-conjugated antibody, while texas red -conjugated-second antibody was used to visualize αLy6G. DAPI stains nuclei. Overlapped staining by both antibodies shows the presence of lactoferrin in the luminal epithelium neutrophils. (B) Immunohistochemistry of serial sections of the uterine lumen revealed that both αLy6G and αLTF antibodies stained these cells in the lumen. Thus, secondary granules of detached neutrophils in the lumen still contain lactoferrin.
(TIF)
