# Supplementary figures and images for: Serine 216 Phosphorylation of Estrogen Receptor α in Neutrophils: Migration and Infiltration into the Mouse Uterus
Source: PLoS One. 2013 Dec 26;8(12):e84462. doi: 10.1371/journal.pone.0084462 (PMC3873424; doi:10.1371/journal.pone.0084462)

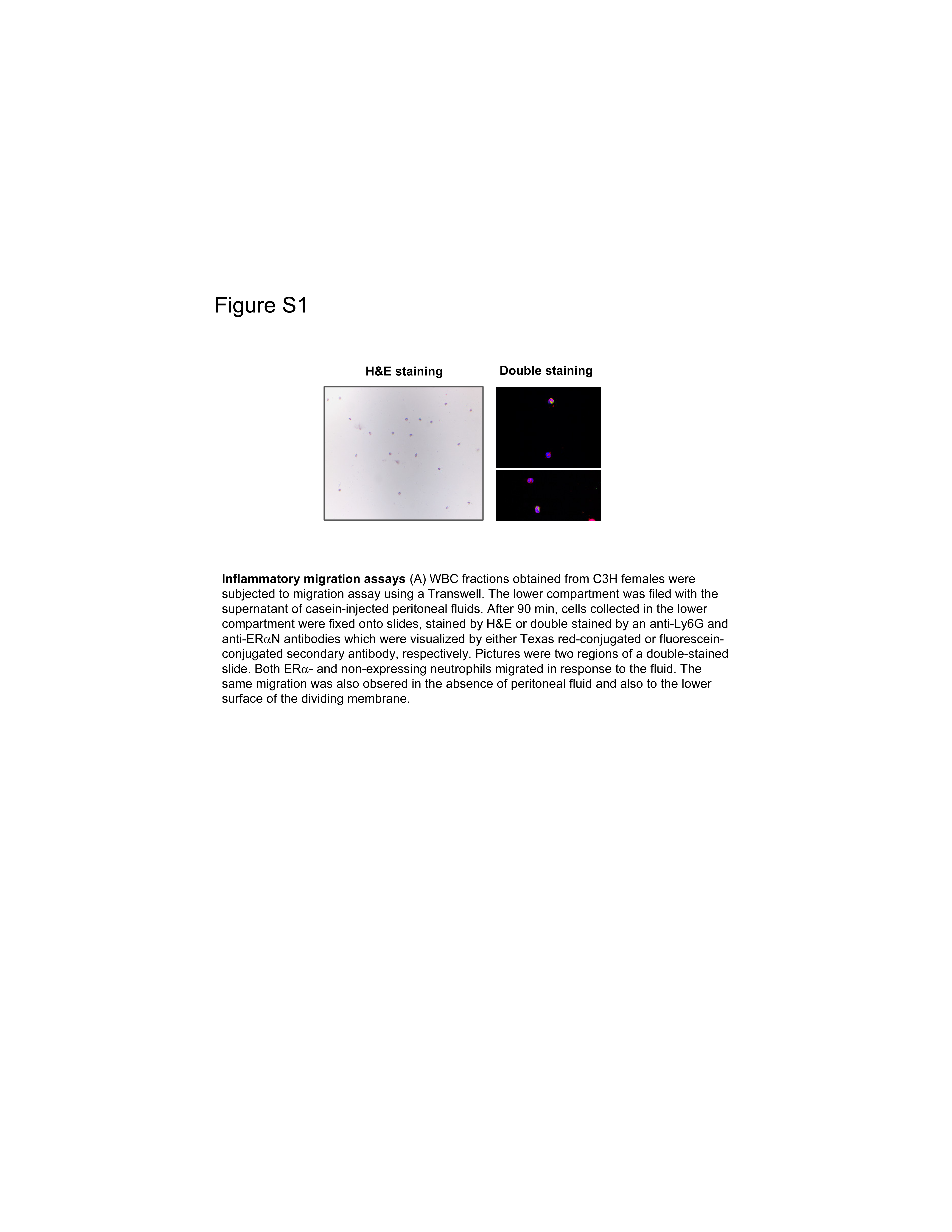

Supplement: Figure S1 — Inflammatory migration assays (A) WBC fractions obtained from C3H females were subjected to migration assay using a Transwell. The lower compartment was filed with the supernatant of casein-injected peritoneal fluids. After 90 min, cells collected in the lower compartment were fixed onto slides, stained by H&E or double stained by an anti-Ly6G and anti-ERαN antibodies which were visualized by either Texas red-conjugated or fluorescein-conjugated secondary antibody, respectively. Pictures were two regions of a double-stained slide. Both ERα- and non-expressing neutrophils migrated in response to the fluid. The same migration was also obsered in the absence of peritoneal fluid and also to the lower surface of the dividing membrane. (TIF) [file pone.0084462.s001.tif]

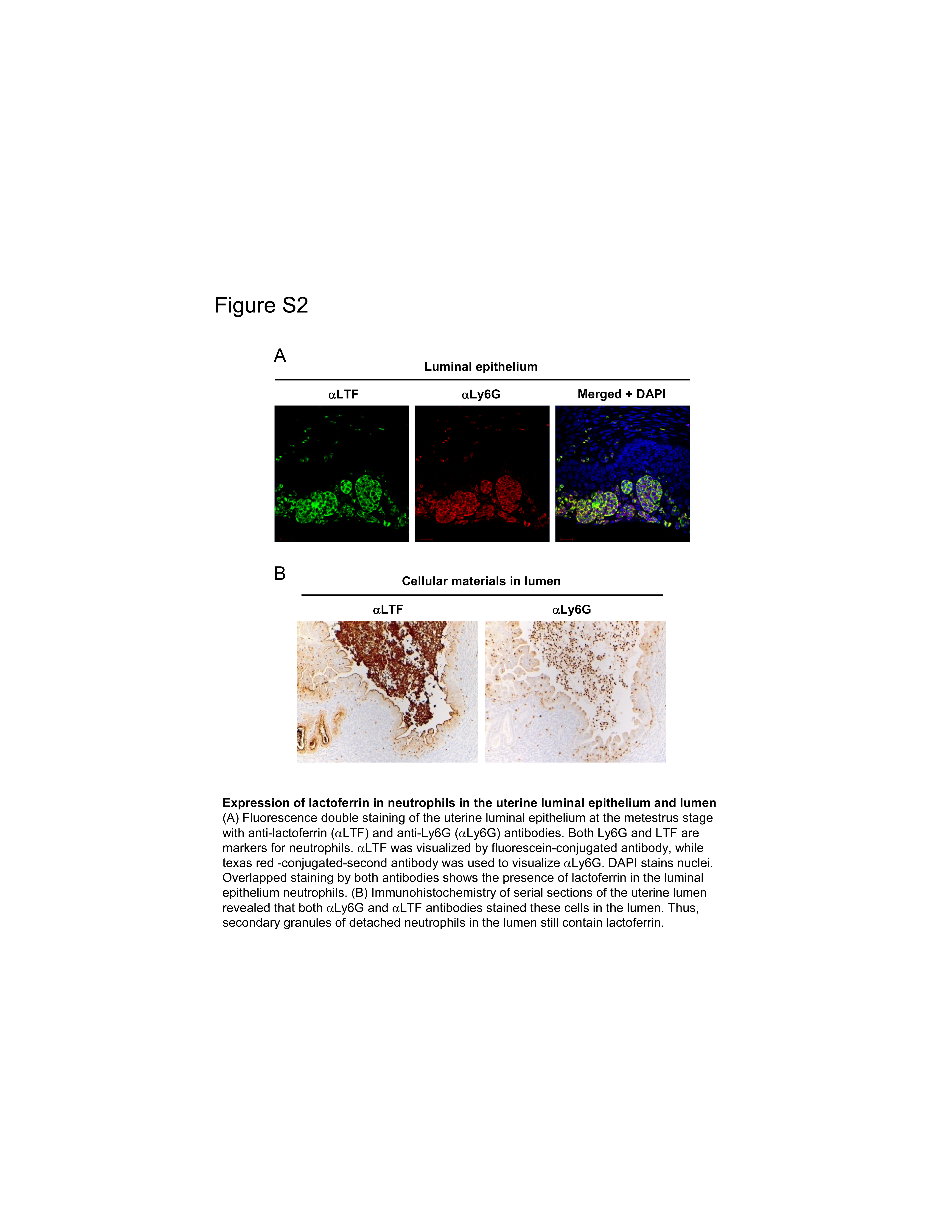

Supplement: Figure S2 — Expression of lactoferrin in neutrophils in the uterine luminal epithelium and lumen (A) Fluorescence double staining of the uterine luminal epithelium at the metestrus stage with anti-lactoferrin (αLTF) and anti-Ly6G (αLy6G) antibodies. Both Ly6G and LTF are markers for neutrophils. αLTF was visualized by fluorescein-conjugated antibody, while texas red -conjugated-second antibody was used to visualize αLy6G. DAPI stains nuclei. Overlapped staining by both antibodies shows the presence of lactoferrin in the luminal epithelium neutrophils. (B) Immunohistochemistry of serial sections of the uterine lumen revealed that both αLy6G and αLTF antibodies stained these cells in the lumen. Thus, secondary granules of detached neutrophils in the lumen still contain lactoferrin. (TIF) [file pone.0084462.s002.tif]
